# Intravesical *Mycobacterium brumae* triggers both local and systemic immunotherapeutic responses against bladder cancer in mice

**DOI:** 10.1038/s41598-018-33253-w

**Published:** 2018-10-10

**Authors:** Estela Noguera-Ortega, Rosa M. Rabanal, Elisabet Gómez-Mora, Cecilia Cabrera, Marina Luquin, Esther Julián

**Affiliations:** 1 Departament de Genètica i de Microbiologia, Facultat de Biociències, Universitat Autònoma de Barcelona, 08193 Bellaterra, Barcelona Spain; 2 Unitat de Patologia Murina i Comparada, Departament de Medicina i Cirurgia Animals, Facultat de Veterinària, Universitat Autònoma de Barcelona, 08193 Bellaterra, Barcelona Spain; 3 AIDS Research Institute IrsiCaixa, Institut de Recerca en Ciències de la Salut Germans Trias i Pujol (IGTP), Hospital Germans Trias i Pujol, Universitat Autònoma de Barcelona, 08916, Badalona, Barcelona, Catalonia Spain

## Abstract

The standard treatment for high-risk non-muscle invasive bladder cancer (BC) is the intravesical administration of live *Mycobacterium bovis* BCG. Previous studies suggest improving this therapy by implementing non-pathogenic mycobacteria, such as *Mycobacterium brumae*, and/or different vehicles for mycobacteria delivery, such as an olive oil (OO)-in-water emulsion. While it has been established that BCG treatment activates the immune system, the immune effects of altering the mycobacterium and/or the preparation remain unknown. In an orthotopic murine BC model, local immune responses were assessed by measuring immune cells into the bladder and macromolecules in the urine by flow cytometry and multiplexing, respectively. Systemic immune responses were analyzed by quantifying sera anti-mycobacteria antibody levels and recall responses of *ex vivo* splenocytes cultured with mycobacteria antigens. In both BCG- and *M. brumae*-treated mice, T, NK, and NKT cell infiltration in the bladder was significantly increased. Notably, T cell infiltration was enhanced in OO-in-water emulsified mycobacteria-treated mice, and urine IL-6 and KC concentrations were elevated. Furthermore, mycobacteria treatment augmented IgG antibody production and splenocyte proliferation, especially in mice receiving OO-in-water emulsified mycobacteria. Our data demonstrate that intravesical mycobacterial treatment triggers local and systemic immune responses, which are most significant when OO-in-water emulsified mycobacteria are used.

## Introduction

Bladder cancer (BC) is the 7^th^ most common cancer in men worldwide^1^. At the time of diagnosis, the tumor is typically confined to the mucosa layer and is called non-muscle invasive BC (NMIBC). After resection of the tumor, for those classified as having a high risk of recurrence and progression, the recommended treatment is the instillation of *Mycobacterium bovis* bacillus Calmette-Guérin (BCG)^2^. The immune system activation by the mycobacterium is key for this therapy to prevent recurrence and progression of the disease, however, the detailed mechanism of action is not completely understood^3^.

The bladder is the perfect cavity to allow close contact between a high concentration of bacilli and epithelial and resident immune cells. Thus, the presence of BCG in the bladder induces the local immune response which involves the release of cytokines and chemokines, initiating a critical immune cascade of events (detailed in excellent reviews)^4–6^. Data from both mice models^7^ and BCG-treated patients^8^ showed that the infiltration of different kinds of immune cells is crucial for tumor clearance in the bladder^9^. Important constituents of the cellular inflammatory response to BCG include CD4^+^ and CD8^+^ lymphocytes, natural killer (NK) cells, and granulocytes^3^. In fact, infiltration of T cells is not observed when heat-killed BCG is instilled into mouse bladders^7^, suggesting that heat-killed BCG cannot replace live BCG to treat BC patients. The systemic immune reaction after the intravesical instillation of BCG is less well understood, although previous studies have shown that cellular and humoral systemic responses could have a role in or be used as markers of the efficacy of BCG therapy^7,10^.

We have recently proposed different strategies to improve both the safety and efficacy of BC treatment: the use non-pathogenic mycobacteria, such as *Mycobacterium brumae* and the use of an improved vehicle to instill the mycobacteria into the bladder. Regarding the former strategy, *M. brumae* showed antitumor activity against bladder cells in *in vitro*, *ex vivo* and *in vivo* assays in an orthotopic model of the disease, which would a safer alternative for BCG treatment^11^. Regarding the vehicle, formulating mycobacteria in an olive oil (OO)-in-water emulsion provided a homogeneous suspension of mycobacteria, avoiding the formation of aggregates that could interfere with the interaction between mycobacteria and immune cells^12^. OO-in-water mycobacteria also have proper physico-chemical properties that potentially facilitate close contact between mycobacteria and the epithelium^12^.

These new options for treatment, including the use of *M. brumae* and possibility of formulating both BCG and *M. brumae*, provide an excellent opportunity to understand how the mycobacteria-induced immune system helps to combat bladder tumors and how the responses differ between mycobacteria treatments and/or the role of the vehicle, if any. For the first time, we will examine the entire immune response triggered by these treatments in an orthotopic model of the disease.

## Results

### Treatment with mycobacteria triggers influx of immune cells into mouse bladders, which was higher when mycobacteria were emulsified

Immune cell infiltration into the bladder, one week after the last treatment, was analyzed by flow cytometry (gating strategy Supplementary Fig. 1). After gating lymphocytes by morphological parameters, only living lymphocytes (defined as AquaDead^−^CD45^+^ cells) were selected. B cell (CD19^+^), NK cell (CD3^−^NK1^+^), NKT cell (CD3^+^NK1^+^) and T cell (CD3^+^CD4^+^ and CD3^+^CD8^+^) subsets were analyzed.

**Figure 1:**
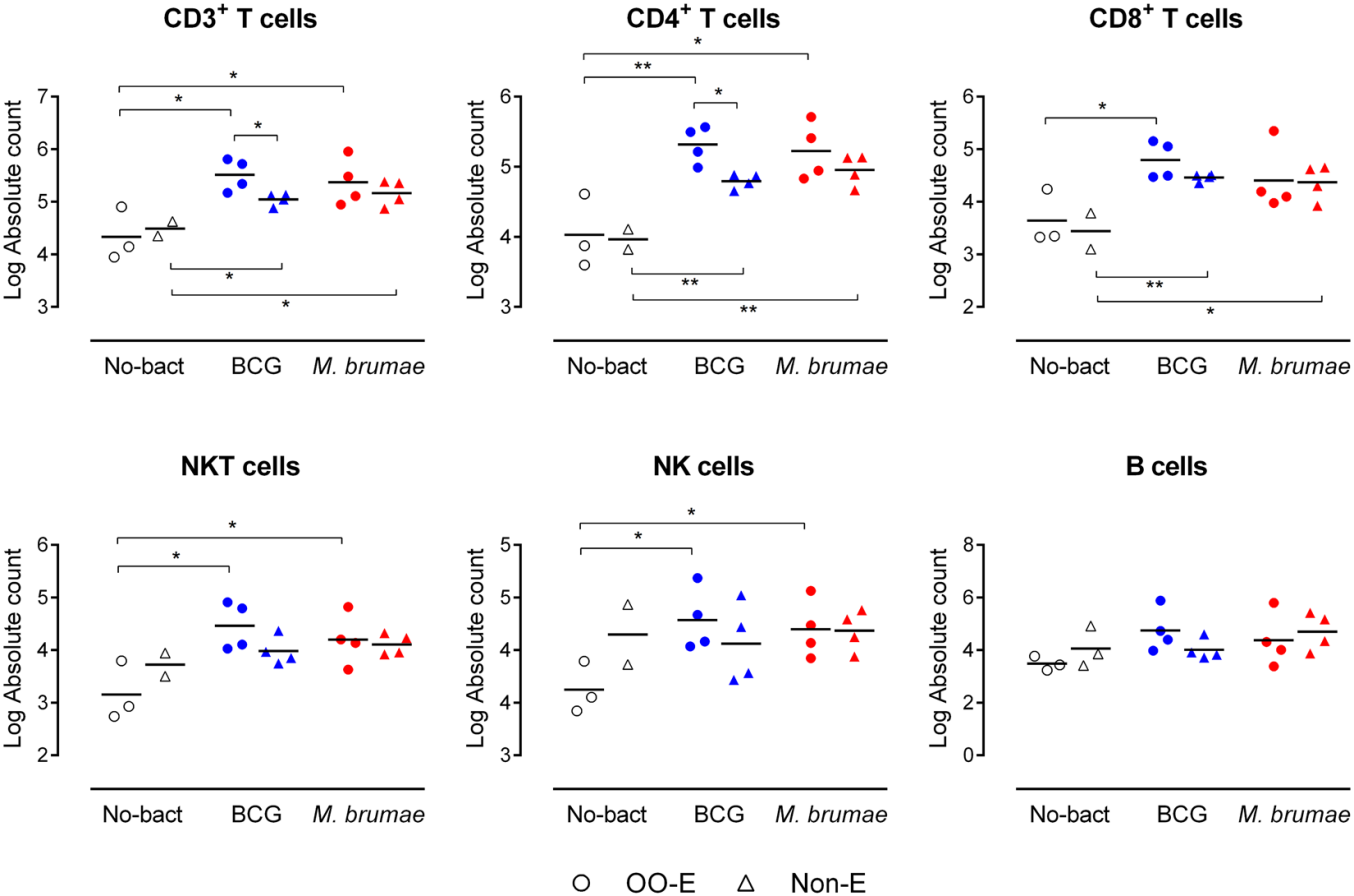
Infiltrated immune cells into mycobacteria-treated and non-treated tumor bearing mice 29 days after tumor induction. Absolute number of T cells (CD3^+^, CD4^+^ and CD8^+^ subsets), NKT cells, NK cells and B cells that infiltrated the bladder. The different groups of animals are indicated using different colors on the horizontal axis of the graph: non-mycobacteria treated mice (No-bact) are represented by empty black symbols, BCG-treated mice by blue symbols and *M. brumae*-treated mice by red symbols. Dots represent animals treated with emulsion or emulsionated mycobacteria, triangles represent mice treated with PBS or mycobacteria in PBS. *p < 0.05; **p < 0.01.

The results show that all mycobacteria treatments induced the infiltration of lymphocytes into the bladder (Fig. 1). Significant differences in the absolute number of infiltrated T cells (both CD4^+^ and CD8^+^ T cells) between controls and mycobacteria-treated mice regardless of the vehicle used were observed (Fig. 1). When treatments were compared among them, a higher infiltration of immune cells in bladders from OO-E treated animals than in the Non-E treated animals was observed, and the number of total CD3 and CD4 subset in BCG-treated mice was significant (Fig. 1). The infiltration of NK and NKT cells was also demonstrated after mycobacteria treatment. Concerning these cells, significant differences between non-treated and mycobacteria-treated mice were found only when OO-E mycobacteria were intravesically instilled, for both BCG and *M. brumae* treatments (Fig. 1). Finally, no differences in infiltrated B cells were found between non-treated mice and BCG or *M. brumae*-treated mice, as shown in Fig. 1.

**Figure 2:**
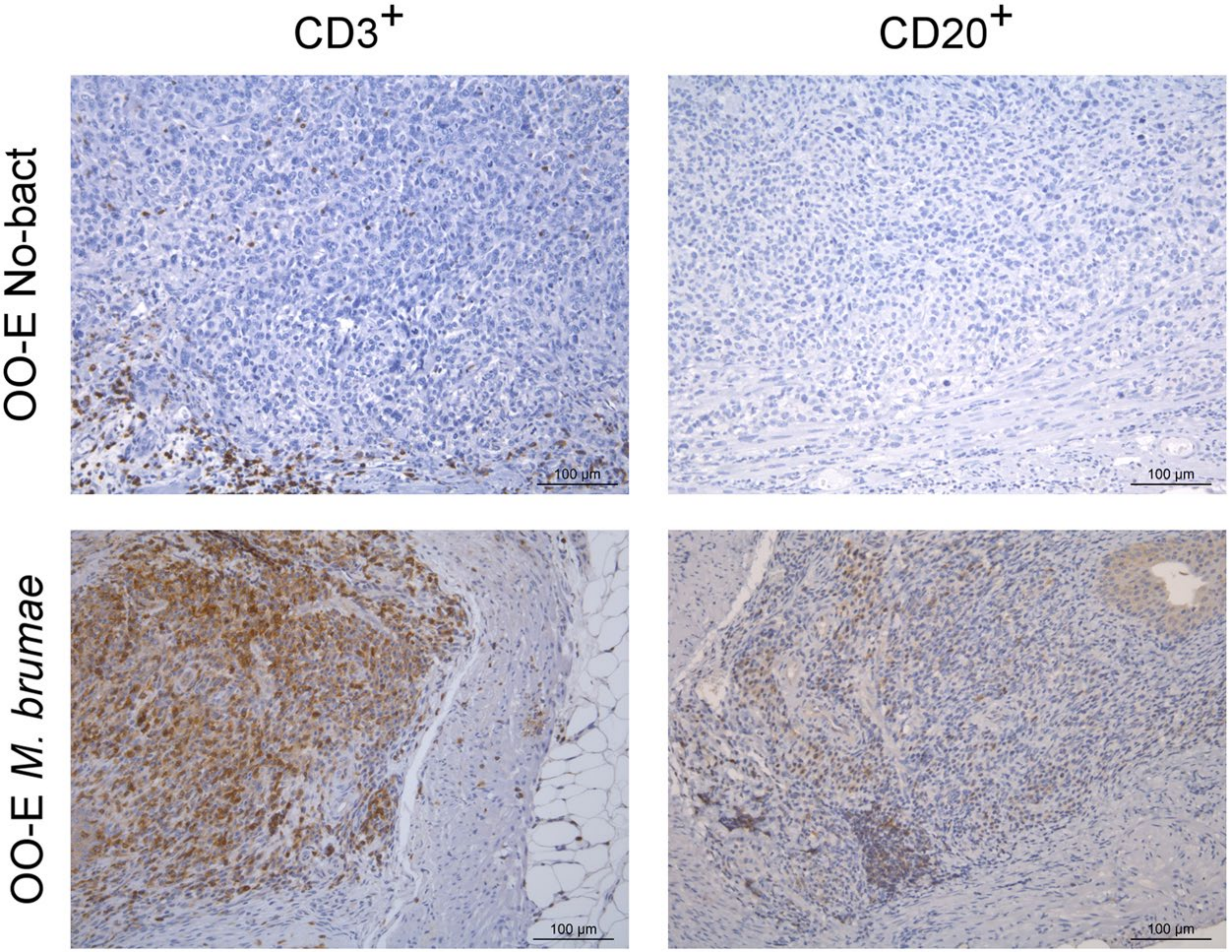
Representative histological images of bladder sections stained with two immunological markers. Bladders from the OO-E No-bact group (top) and from the OO-E live *M. brumae* group (bottom). Sections were stained with a CD3 marker (left column) and CD20 marker (right column). Scale bar, 100 µm.

The IHQ findings confirmed the results obtained by cytometry analysis. First, we observed that the presence of tumors inside the bladders triggered the infiltration of some CD3^+^ cells and few CD20^+^ cells (B cells). The treatment of tumor-bearing mice with mycobacteria induced the infiltration of both CD3^+^ and CD20^+^ cells in the lamina propria and in some cases numerous CD3^+^ cells had infiltrated (Fig. 2). Regarding the localization of the populations, the IHQ analysis showed that CD3^+^ cells were generally localized in the peritumoral area in the animals that had tumors, regardless of whether the tumors were diffuse cells or solid masses.

### KC, IL-6, MIP-2, MMP-9 and VEGF are present in urine samples from mycobacteria-treated tumor-bearing mice

Among all of the molecules studied in the urine samples, only IL-6, KC, VEGF, MIP-2 and MMP-9 levels were detected in the groups of studied mice. Other molecules, such as IFN-γ, IP-10, GM-CSF, or TNF-α, were detected only in some groups at determined time-points while the remaining molecules (IL-1β, IL-2, IL-4, IL-5, IL-10, IL-12 p70, IL-13, IL-17, MCP-1 and RANTES) were under the limit of detection of the kit. Although using pooled samples did not allow us to calculate significant differences between treatment groups, a trend was observed, as shown in Supplementary Fig. 2. Regarding IL-6 and KC, an increase in their presence was observed in urine samples from all of the mice and was notably higher in mice treated with emulsified BCG. Regarding VEGF and MIP-2, while healthy mice had low levels at all time-points, a peak of production was detected at day 15 after tumor induction in all of the tumor-bearing mice regardless of whether they received mycobacteria treatment. In general, from day 15 to 29 after tumor induction, the levels of these molecules decreased except in the untreated tumor-bearing mice (No-bact groups). Concerning MMP-9, a general trend was observed in all of the groups, showing a production peak two days after tumor induction and decreasing levels of this molecule were then detected in the following weeks.

### Mycobacteria treatment induces the production of BCG and *M. brumae* specific antibodies

**Figure 3:**
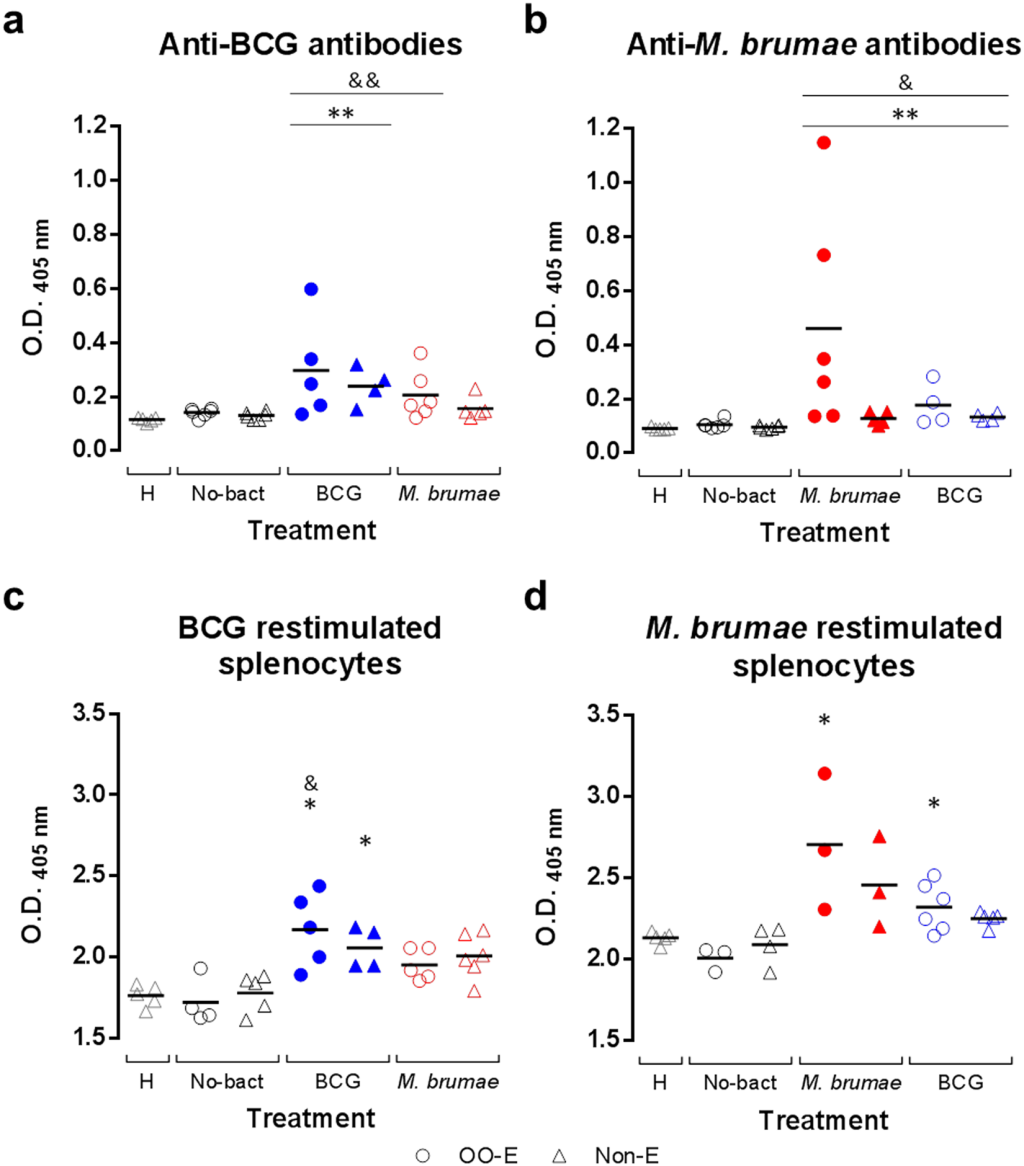
Systemic immune responses in tumor-bearing and healthy mice. Levels of anti-BCG (**a**) and anti-*M. brumae* (**b**) IgG antibodies present in sera from BCG-treated, *M. brumae*-treated and non-treated tumor-bearing mice, as well as in healthy animals. The presence of BCG antibodies in *M. brumae*-treated mouse serum is represented by empty blue symbols (**b**) and the presence of *M. brumae* antibodies in BCG-treated mouse serum by empty red symbols (**a**). For a and b, solid lines represent the mean of the OD (405 nm) values (two technical replicates) of the six mice from each group. *p < 0.05 compared to No-bact. ^&^p < 0.05 compared to H. Splenocyte proliferation after *ex vivo* BCG (**c**) and *M. brumae* (**d**) restimulation. Proliferation is expressed relative to non-restimulated splenocytes of the same animal (proliferation of restimulated splenocytes/proliferation of non-restimulated splenocytes) after BCG and *M. brumae* stimulation. Proliferation of splenocytes from *M. brumae*-treated animals restimulated with BCG antigens is represented by empty red symbols (**c**) and proliferation of splenocytes from BCG-treated animals restimulated with *M. brumae* antigens is represented by empty blue symbols (**d**). For c and d, solid lines represent the mean of relative proliferation (three technical replicates) of 3–6 different spleens from each animal group. *p < 0.05 compared to No-bact. ^&^p < 0.05 compared to H. For a, b, c, and d, dots represent animals treated with emulsified preparations and triangles represent animals treated with non-emulsified preparations. The different groups of animals are indicated by different colors on the horizontal axis of the graph as follows: empty grey triangles for the healthy (H) mice, empty black symbols for tumor-bearing mice receiving no mycobacteria treatment (No-bact), blue symbols for BCG-treated mice, and red symbols for *M. brumae*-treated mice.

When the production of anti-BCG and anti-*M. brumae* IgG antibodies was analyzed in serum samples from BCG-treated and *M. brumae*-treated mice, respectively, significant IgG production in all mycobacteria-treated groups compared to healthy mice and non-treated tumor-bearing mice was observed (Fig. 3a,b). In both BCG- and *M. brumae*-treated mice, higher levels of antibodies were present in sera from animals treated with emulsified mycobacteria than those treated with non-emulsified mycobacteria, although the differences were not significant. High levels of anti-*M. brumae* antibodies were found in *M. brumae*-treated mice compared to the other groups (Fig. 3b). A cross-reaction antibody response between *M. brumae* and BCG was also studied. As shown in Fig. 3a,b, the presence of anti-BCG IgG levels in sera from *M. brumae*-treated mice were detected, and *viceversa*, although the levels were not higher than those detected against the antigen used for treatment. This result suggests the presence of shared antigens among them, as expected, as well as mycobacteria specific antigens. In all groups, the presence of anti-mycobacteria IgA antibodies in sera was insignificant (data not shown).

### Splenocytes from mycobacteria-treated mice specifically proliferate in the presence of the antigens used in the instillations

To demonstrate a systemic immune response to emulsified and non-emulsified mycobacteria after intravesical instillation, splenocyte proliferation from sacrificed mice was measured after *ex vivo* restimulation. First, unspecific proliferation was observed in all cultures after stimulation with Concanavalin A. When splenocytes were stimulated *in vitro* with the same mycobacterium that was used for intravesical instillation, increased proliferation was observed in cultures from mycobacteria-treated mice compared to those from non-treated tumor-bearing mice, which was significant in all cases except for non-emulsified *M. brumae*-treated mice (Fig. 3c,d). Again, an enhanced immune response was observed in splenocytes from emulsified-mycobacteria-treated mice compared to those obtained from non-emulsified mycobacteria-treated mice. When a possible cross-reaction was evaluated, the same trend mentioned for antibody detection was observed: higher proliferation was observed when the same mycobacteria were used for *in vivo* treatment and *in vitro* restimulation (Fig. 3c,d).

## Discussion

This is the first study to investigate the local and systemic immune response of BCG compared to the promising and safe *M. brumae* immunotherapeutic agent. *M. brumae* is an efficacious tool for inhibiting bladder tumor growth both *in vitro* and *in vivo*, both formulated in an OO-in-water emulsion and non-emulsified^11,12^. *M. brumae* is also an excellent immunostimulatory agent by triggering the production of cytokines in BC cells, macrophages and peripheral blood in *in vitro* cultures^11^, but its immunotherapeutic role *in vivo* is unknown.

Our first relevant result is the induction of a massive infiltration of T lymphocytes into the bladder, both CD4^+^ and CD8^+^ T cells in *M. brumae*-treated tumor-bearing mice compared to the basal infiltration that is observed in non-treated tumor-bearing mice. This local immune response is similar to that induced by BCG. When formulated mycobacteria were used, the immune cell infiltrate in the bladder was further increased, which was only statistically significant when CD3^+^ and CD4^+^ T cells were counted. The influx of immune cells into the bladder after treatment is critical in BC patients for a successful BCG treatment. Specifically, an increased intratumoral CD4^+^ T cell population has been significantly associated with BCG response and longer recurrence-free survival in BC patients^8^. The orthotopic murine model of the disease is a good model to evaluate this effect. Mice depleted of CD4 or CD8 populations showed decreased survival rates after tumor induction compared to immunocompetent mice. Other than T cells, the remainder of the lymphocyte subset analysis also showed significant amounts of NK and NKT cell bladder infiltration when emulsified *M. brumae* or BCG was used for intravesical treatment. Brandau *et al*.^13^ also demonstrated that the BCG-induced presence of NK cells is relevant for control of the primary tumor by depleting this cell population in the orthotopic murine model. The fact that only emulsified mycobacteria significantly triggered the infiltration of these NK cells highlights the importance of the vehicle in which the therapeutic agents are instilled into the bladder. These results confirm that the oil-in-water emulsion allows the mycobacteria to reach the epithelium and exert their antitumor effect as we suggested previously^12^.

This profile of cellular infiltration triggered by *M. brumae* could be indicative of a biased favorable Th1 response, as has been described for BCG. BCG responders release higher amounts of Th1 cytokines, such as IFN-γ, IL-12 or IL-2 than Th2 cytokines, such as IL-4, IL-5 or IL-10, in the urine. In contrast, if the response is similar to Th2, the therapy fails^14,15^. We measured the presence of a broad panel of cytokines and chemokines in mouse urine but our results were not conclusive mainly due to the amount of samples we obtained. Only five macromolecules (IL-6, KC, VEGF, MIP-2 and MMP-9) were detected. Repeated instillations of mycobacteria might be responsible for the peak of IL-6 and KC observed at day 15^7,15–17^ and was correlated with the influx of some immune cells detected by flow cytometry and immunohistochemistry (Figs 1 and 2). Furthermore, although IL-6 induces the Th2 response, which might be controlled by the presence of IFN-γ and the absence of IL-4^18^, IL-6 also up-regulates the fibronectin α5β1 receptor, which is involved in the interaction of BCG with the urothelium^19,20^, which inhibits bladder tumor cell proliferation. It is worth mentioning, our previous results showed that OO-in-water emulsified *M. brumae* has a higher affinity for fibronectin than non-emulsified mycobacteria^12^.

The second relevant result was the induction of a systemic, both humoral and cellular, immune response by *M. brumae* instillation, which was also enhanced when emulsified mycobacteria was used for treatment (Fig. 3). To the best of our knowledge, this is the first work that demonstrates a specific antibody production against *M. brumae* or BCG in the orthotopic murine model of the disease. Previous studies have demonstrated the presence of circulating anti-BCG antibodies in BC patients, with uncertainty on whether their presence correlates with a positive outcome^21,22^ of the treatment or whether it has any predictive value^23,24^. Apart from its use as a predictive biomarker for mycobacteria treatment in BC patients, whether the induction of this humoral response has a role in mycobacteria-induced immunotherapy requires further investigation. The orthotopic model can then be a useful tool. The other relevant result related to the systemic response was that the non-pathogenic mycobacterium *M. brumae* or *M. brumae*-derived antigens, could reach the spleen of treated mice as specific proliferation after restimulation (Fig. 3), and IFN-γ production^12^ was observed in cultured splenocytes from *M. brumae*-treated mice, identifying then memory immune cells in the spleen^15^. The synthesis of IFN-γ and the fact that IL-4 was not detected^12^, suggest that, although *M. brumae* elicits both humoral (Th2) and cellular (Th1) immune responses, this response was mainly biased to a Th1 response, as was the response to BCG^14,25^. Unlike BCG, which was isolated from splenocytes of BCG-treated tumor-bearing mice even four weeks after the last instillation, *M. brumae* was not found in the spleen cells one week after the last instillation, as we have previously shown in this orthotopic mouse model^11,26^. One possibility may be that *M. brumae* reached the bloodstream in treated tumor-bearing mice and arrived at the spleen, like BCG, and was rapidly cleared. The ability of *M. brumae* to reach different organs after infection in healthy mice is currently being studied to elucidate the mechanism of its immunomodulatory capability. Moreover, our results shed light on another interesting fact. An expected cross-reaction is observed when sera or splenocytes from BCG-treated mice are in contact with *M. brumae* antigens and *viceversa* (Fig. 3), due to the presence of common mycobacteria antigens. However, although not significant, a trend was observed towards specificity in this cross-reactive response. Interestingly, cross-reactive stimulation of splenocytes resulted in no IFN-γ production in supernatant cultures by these cells (data not shown), indicating that it is necessary to restimulate with the same antigens with which the animals were treated to trigger IFN-γ production, as observed with the splenocyte proliferation. Thus, both humoral and restimulatory reactions suggest that some *M. brumae*- or BCG-specific antigens could be implicated in the systemic immune response.

Despite known limitations, such as using exclusively female mice or the variability of tumor cells implanted into the bladder^27^, which could also lead to variability in the immune response generated by mycobacteria treatment, the orthotopic murine BC model is a solid, well-recognized model that provides consistent results to evaluate the immune response triggered by potential new antitumor therapies, such as IL-12 plus chitosan^28^, or the synergy of different treatments^29^. However, one drawback of our study is the low number of mice that survived until day 29 after tumor induction in the non-treated group of animals due to the high mortality rates in this group. It has previously been demonstrated that at least three to four BCG weekly instillations are needed in healthy mice to observe the infiltration of immune cell populations into the bladder, reaching a peak 28–30 days after initiating the treatment^7^. More animals in the non-treated tumor-bearing mouse group could provide significant differences between mycobacteria-treated and non-treated mice in regard to some of the immune parameters analyzed. In view of our results, further investigation is warranted to elucidate the complete immune response (other immune subsets of cells or local and systemic immune biomarkers) induced by *M. brumae* compared to BCG to completely understand the role of mycobacteria in modulating the tumor microenvironment. Different strategies for superficial bladder treatment are currently being proposed: diverse bacteria^30–32^, modified virus^33,34^, chemotherapeutic agents^35^, molecules that target specific immune receptors^36^, peptide vaccines^37^, plant derived compounds^38^, etc. Mycobacteria can have a role to synergy to these treatments, as has been already demonstrated^35,37,38^, enabling to reduce the toxicity and resistance to a single treatment. Further understanding of the immune reaction associated with mycobacteria treatment could be critical for identifying the benefits of new immunotherapeutic possibilities.

In summary, we showed that *M. brumae* triggers selective infiltration of immune cells into the bladder similar to BCG, inducing a favorable systemic immune response in treated tumor-bearing mice.

## Material and Methods

### Bacterial strains and cell line

*M. bovis* BCG Connaught (ATCC 35745) and *M. brumae* (ATCC 51384^TM^) were grown on Middlebrook 7H10 medium (Difco Laboratories, Surrey, UK) supplemented with 10% oleic-albumin-dextrose-catalase enrichment, for 4 or 1 week, respectively, at 37 °C. For *in vitro* stimulation of splenocytes and for coating enzyme-linked immunosorbent assay (ELISA) plates, suspensions of *M. brumae* or BCG were heat-killed at 121 °C for 30 minutes^39^. Mycobacteria emulsified in OO (Sigma) were prepared as previously described^12^. For tumor induction, MB49 BC murine cells were cultured as previously described^11,26^.

### Orthotopic murine model of BC and intravesical treatment

**Figure 4:**
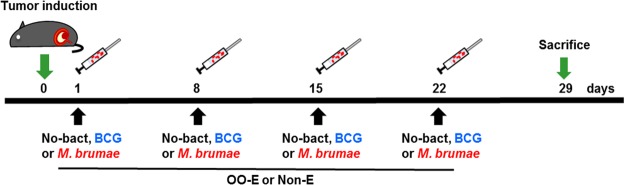
Schedule of the animal experiments. Graphical representation of the schedule, in which tumor induction (day 0), mycobacteria treatments (days 1, 8, 15 and 22 after tumor induction) and sacrifice (day 29 after tumor induction) are indicated by arrows.

Animal experiments were performed according to procedures approved by the Animal Care Committee at the Universitat Autònoma de Barcelona. The orthotopic murine model of BC was developed as previously described^11,26^. Briefly, six C57Bl/6 female mice (6–8 weeks old; Charles River Laboratories, France) were anaesthetized and chemical lesions were induced by intravesical instillation of L-poly-lysine (Sigma). Subsequently, 10^5^ MB49 bladder tumor cells were instilled to induce tumors. One day later, each group of mice received intravesically 100 µl of mycobacteria suspensions (corresponding to 2 × 10^6^ colony forming units (CFU) of BCG or 2 × 10^7^ CFU of *M. brumae* per mouse) or vehicle for control groups. Animals were treated weekly following the schedule shown in Fig. 4 and were sacrificed at day 29 after tumor induction. At the time of sacrifice, blood was collected and bladders and spleens aseptically removed. Bladders from some of the animals of each group were processed for immunohistochemistry (IHQ) analysis, and the other bladders for cytometry studies.

### Detection of infiltrated immune cell subsets in bladders

For flow cytometry experiments, bladders were minced using a scalpel followed by digestion with Roswell Park Memorial Institute (RPMI) medium supplemented with 5% fetal bovine serum (FBS) containing 0.5 mg/mL collagenase II (Sigma, Spain) at 37 °C for two successive 30 min cycles, with continuous shaking. The cell suspension obtained was consecutively filtered through a 70-μm disposable plastic strainer (Becton & Dickinson) and pelleted for staining. The cells were labeled with the following antibodies: PerCP-CD45 and PECy7-NK1.1, purchased from Biolegend, FITC-CD3, PE-CD4, APC-CD8, PE-CD19 and APC-CD11b, from Immunotools. The lymphocyte gate was defined by morphological parameters, and dead cells were excluded using a live/dead fixable Aqua Dead Cell Stain kit (Invitrogen). Samples were acquired in a LSRII flow cytometer (Becton & Dickinson), and the data were analyzed using FlowJo software (9.8 v; TreeStar, Portland, OR, USA). Absolute cell numbers were obtained by using Perfect-Count Microspheres (Cytognos).

For IHQ, 3-µm paraffin-embedded tissue sections were dewaxed and endogenous peroxidase activity blocked by exposure to 3% H_2_O_2_ for 40 min. For antigen retrieval, sections were treated with 0.1% protease (Sigma) in PBS for 8 min at 37 °C (CD3) or boiled in bain-marie at 96–98 °C for 20 min in 0.01 M citrate buffer (pH 6.0) (CD20). Non-specific protein binding was blocked with normal goat serum (X0907, Dako) or normal rabbit serum (X0902, Dako) for 1 h at room temperature (RT). T-cell and B-cell immunolabeling was performed using a polyclonal rabbit anti-human CD3 antibody (Dako) diluted 1:300 or CD20/ MS4A1 antibody (Thermo Scientific™ Pierce™) diluted 1:200, respectively, overnight (ON) at 4 °C. The positive control tissue consisted of sections of normal mouse lymph node. An EnVision^TM^+ System-HRP Rabbit Kit (Dako) was used to detect CD3 and CD20. The chromogen substrate was 3,3′-diaminobenzidine (Dako). The sections were counterstained with hematoxylin (Merck). Negative control sections from the same specimens were identically processed, replacing the specific primary antibody with an isotype-control IgG of the same species and concentration as the primary antibody.

### Detection of macromolecules in urine samples

To detect macromolecules, pools of urines from each group of animals were collected at different time-points: day 0, before tumor induction; day 1, before the first dose of treatment; days 2 and 15, 24 h after the intravesical treatment; and day 29, before sacrificing the animals. Collected urine samples were centrifuged, stabilized in stabilization buffer^15^ and frozen at −40 °C. Thawed urine samples were diluted 1:2 in Calibrator Diluent RD6-52 (R&D Biosystems) and analyzed for the presence of mouse granulocyte–macrophage colony-stimulating factor (GM-CSF), interferon (IFN)-γ, IFN-γ-inducible protein-10 (IP-10), interleukin (IL)-1β, IL-2, IL-4, IL-5, IL-6, IL-10, IL-12 p70, IL-13, IL-17, keratinocyte chemoattractant (KC), macrophage inflammatory protein-2 (MIP-2), matrix metaloproteinase-9 (MMP-9), monocyte chemoattractant protein-1 (MCP-1), regulated on activation, normal T cell expressed and secreted (RANTES), tumor necrosis factor-α (TNF-α), and vascular endothelial growth factor (VEGF) using a multiplex kit (R&D Biosystems). The assay was performed following the manufacturer’s instructions. The plate was read using Luminex® Magpix® equipment. xPONENT® 4.2 software was used for quantitative data acquisition and Milliplex® Analyst 5.1 for multiplex data analysis.

### Detection of anti-mycobacteria antibodies in sera

Mice were bled by cardiac puncture when sacrificed; sera were collected after blood centrifugation and kept at −40 °C until use. ELISA plates (Costar) were coated with either heat-killed BCG or *M. brumae* cells (20 µg/mL) in carbonate–bicarbonate buffer (pH 9.6) ON at RT^40,41^. After washing with Tris-buffered saline (TBS), the wells were blocked with 0.5% gelatin (Sigma) in TBS (blocking buffer) for 2 h^42^. After 3 washes, serum samples were diluted 1/50 in blocking buffer for IgG and without dilution for IgA detection and were incubated ON at RT. The plates were then washed and incubated for 1 h at RT with alkaline phosphatase-labeled goat anti-mouse IgG or IgA antibodies (Southern Biotech, Birmingham, AL, USA) diluted in blocking buffer. The enzyme–substrate reaction was developed using p-nitrophenyl phosphate (Sigma), and the absorbance was measured in a multiscan reader at 405 nm (Tecan). Blank wells (without antigen) were included for each sample, and the absorbance values were deducted from those obtained in antigen-coated wells.

### Proliferation of mycobacteria-stimulated splenocyte cultures

Spleens were individually disrupted in SensiCell^TM^ RPMI medium 1640 with glucose, sodium pyruvate and stable glutamine (Life Technologies) supplemented with 10% FBS (complete medium). Splenocyte suspensions were allowed to settle at 4 °C for at least 30 min. Supernatants were collected and the cell concentration was adjusted to 3 × 10^6^ cells/mL in complete RPMI medium with penicillin (100 U/mL) (ERN, Spain) and streptomycin (100 µg/mL) (Reig Jofre, Spain). One hundred microliters was added to 96-well flat-bottomed microtiter plate (Nunc, Denmark) wells with or without 1 mg/mL of heat-killed BCG or *M. brumae* cells or 5 µg/mL of Concanavalin A (ConA, Sigma) as a positive control. After incubation for 72 hours at 37 °C in 5% CO_2_, supernatants were collected, and cell proliferation was measured using a 3-(4,5-dimethylthiazol-2-yl)-2,5-diphenyltetrazolium bromide (MTT) assay as described previously^43,44^.

### Statistical analysis

A *t-test* was used to compare flow cytometry data and the proliferation of splenocytes (GraphPad Prism). Immunoglobulin levels in sera samples were compared using a Mann-Whitney U test using GraphPad Prism software. Significance was defined as p < 0.05.

## Electronic supplementary material

Supplementary Figures

## Data Availability

All data generated or analysed during this study are included in this published article (and its Supplementary Information files).
