# Supplementary material for: Intravesical Mycobacterium brumae triggers both local and systemic immunotherapeutic responses against bladder cancer in mice
Source: Sci Rep. 2018 Oct 10;8:15102. doi: 10.1038/s41598-018-33253-w (PMC6180069; doi:10.1038/s41598-018-33253-w)
Supplement: Supplementary file 1 — Supplementary Figures [file 41598_2018_33253_MOESM1_ESM.pdf]

Intravesical *Mycobacterium brumae* triggers both local and systemic immunotherapeutic responses against bladder cancer in mice

Estela Noguera-Ortega[1], Rosa M. Rabanal[2], Elisabet Gómez-Mora[3], Cecilia Cabrera[3], Marina Luquin[1], and Esther Julián[1,*]

[1]Departament de Genètica i de Microbiologia, Facultat de Biociències, Universitat Autònoma de Barcelona, 08193, Bellaterra, Barcelona, Spain.

[2]Unitat de Patologia Murina i Comparada, Departament de Medicina i Cirurgia Animals, Facultat de Veterinària, Universitat Autònoma de Barcelona, 08193, Bellaterra, Barcelona, Spain.

[3]AIDS Research Institute IrsiCaixa, Institut de Recerca en Ciències de la Salut Germans Trias i Pujol (IGTP), Hospital Germans Trias i Pujol, Universitat Autònoma de Barcelona, 08916, Badalona, Barcelona, Catalonia, Spain.

*****Correspondence: E-mail: esther.julian@uab.cat; Tel.: (+34) 93 581 4870

Supplementary Figure 1. Gating strategy to analyze the quantity and phenotype of immune cells that infiltrated the bladders.

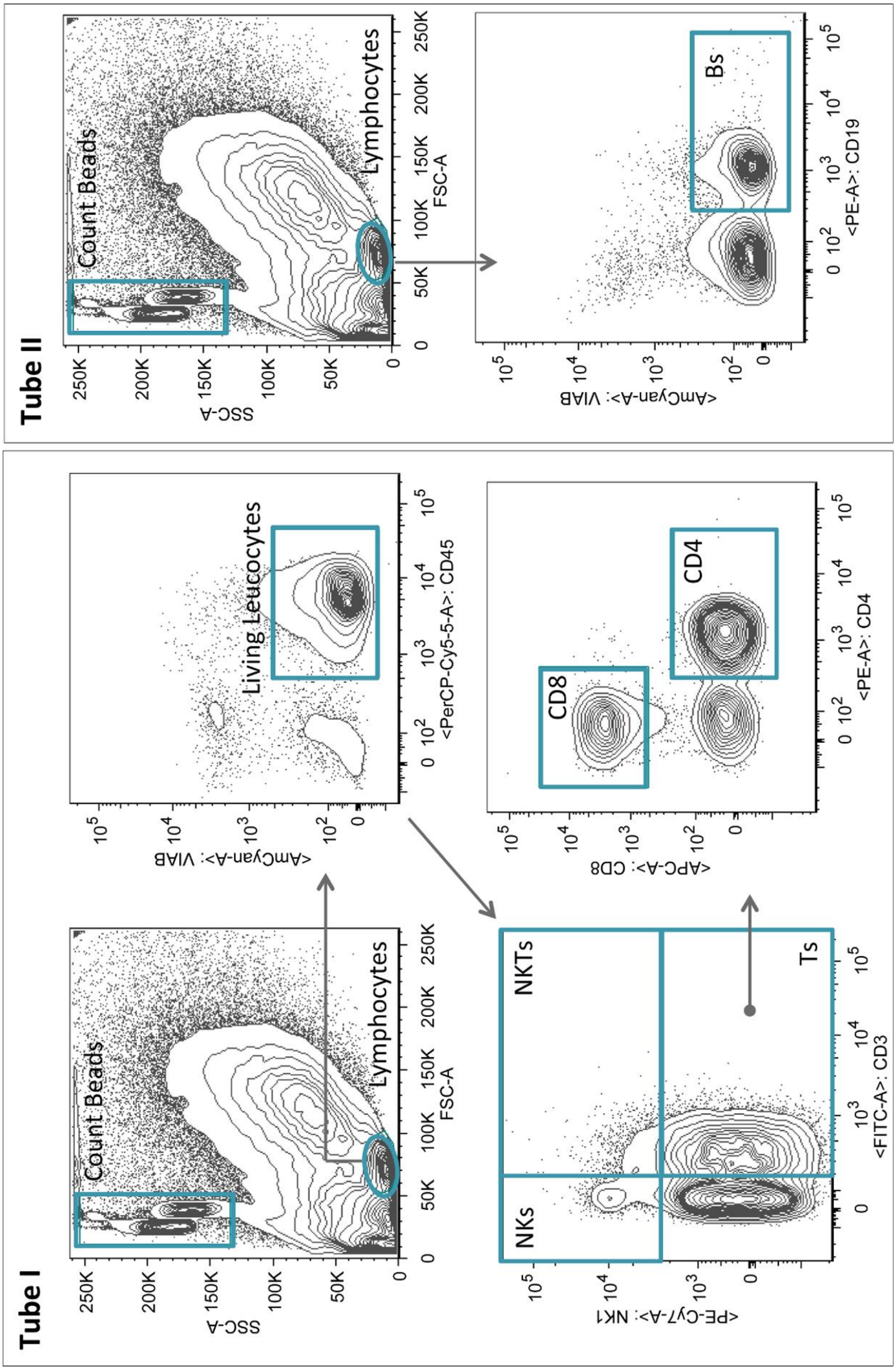

Supplementary Figure 2. Macromolecules detected in pooled urine taken at different time-points from mice.

IL-6, KC, VEGF, MIP-2 and MMP-9 were detected in pooled urine samples taken from mice at different time points. Dots plus solid lines represent tumor-bearing mice treated with emulsified preparations and triangles plus dotted lines represent animals treated with non-emulsified preparations. The different groups of animals are represented by different colors: grey triangles represent the healthy (H) mice, tumor-bearing-mice not receiving mycobacteria treatment (No-bact) are represented by black symbols, BCG-treated mice by blue symbols and *M. brumae*-treated mice by red symbols.

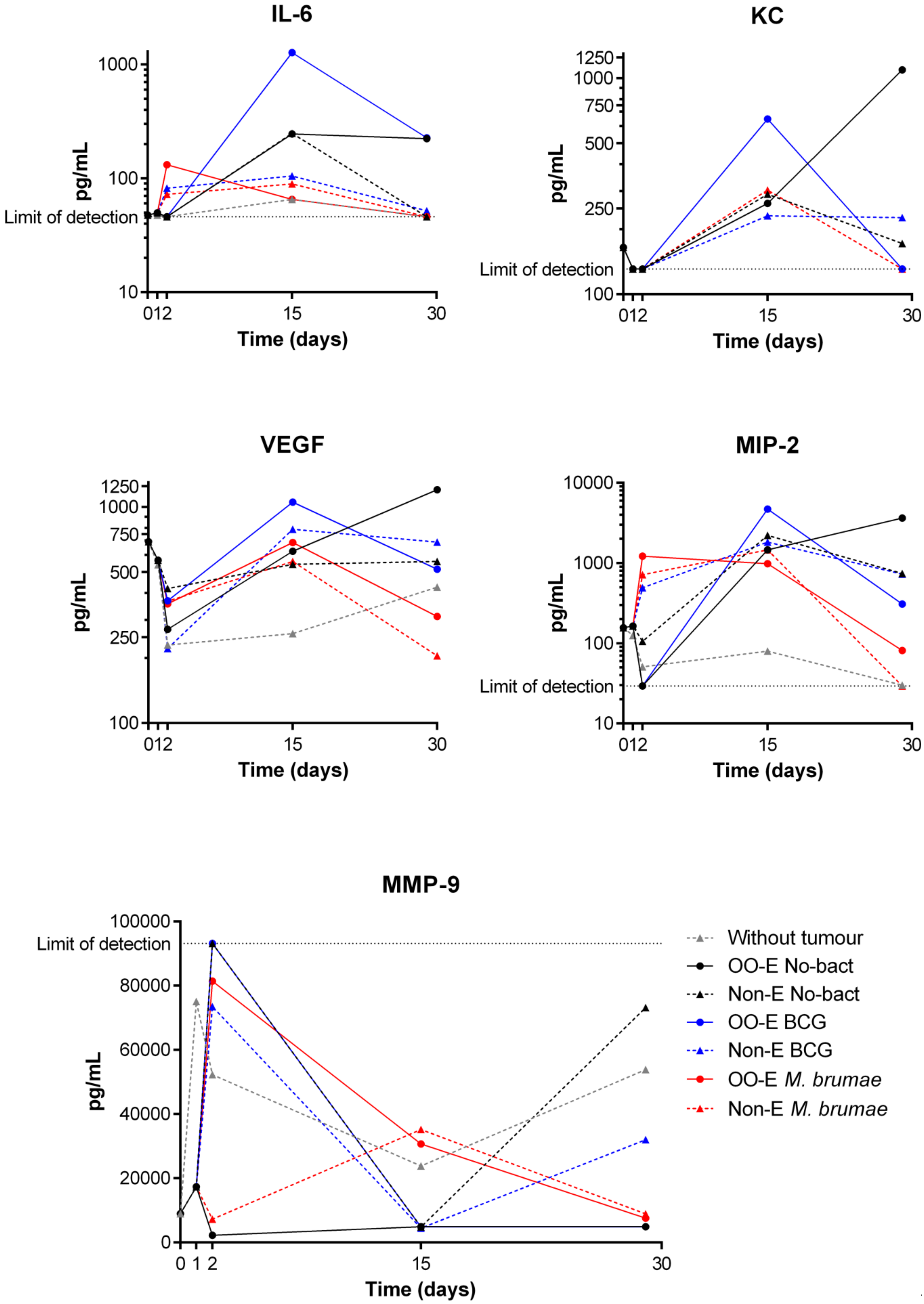
IL-6
KC
VEGF
MIP-2
MMP-9
pg/mL
Limit of detection
Time (days)
0 1 2
15
30
1000
100
10
1250
750
500
250
10000
100000
80000
60000
40000
20000
0
Without tumour
OO-E No-bact
Non-E No-bact
OO-E BCG
Non-E BCG
OO-E *M. brumae*
Non-E *M. brumae*
